# Design and preparation of Fe_3_O_4_@PVA polymeric magnetic nanocomposite film and surface coating by sulfonic acid via in situ methods and evaluation of its catalytic performance in the synthesis of dihydropyrimidines

**DOI:** 10.1186/s13065-019-0538-2

**Published:** 2019-02-04

**Authors:** Ali Maleki, Maryam Niksefat, Jamal Rahimi, Zoleikha Hajizadeh

**Affiliations:** Catalysts and Organic Synthesis Research Laboratory, Department of Chemistry, Iran University of Science and Technology, Tehran, 16846-13114 Iran

**Keywords:** Polyvinyl alcohol, Magnetic nanocomposite film, Heterogeneous nanocatalyst, Dihydropyrimidinone, Green chemistry

## Abstract

For the first time, the design and preparation of magnetic polyvinyl alcohol (Fe_3_O_4_@PVA) nanocomposite film as a novel nanocatalyst was accomplished by in situ precipitation method. To enhance the catalysis activity, the surface modification of this nanocomposite was carried out by sulfonic acid. After the synthesis of this nanocomposite film, Fourier-transform infrared (FT-IR) spectroscopy, energy-dispersive X-ray (EDX) analysis, field-emission scanning electron microscopy (FE-SEM), transmission electron microscopy (TEM) images, X-ray diffraction (XRD) pattern, N_2_ adsorption–desorption by Brunauer–Emmett–Teller (BET), thermogravimetric analysis (TGA) and vibrating sample magnetometer (VSM) were utilized to confirm the structure of the nanocomposite. The catalytic activity of Fe_3_O_4_@PVA was investigated by the synthesis of dihydropyrimidine derivatives from an aldehyde, ß-ketoester and urea or thiourea. This heterogeneous nanocatalyst can be easily separated by an external magnet and reused for several times without any significant loss of activity. Simple work-up, mild reaction conditions and easily recoverable catalyst are the advantageous of this nanocomposite film.

**Electronic supplementary material:**

The online version of this article (10.1186/s13065-019-0538-2) contains supplementary material, which is available to authorized users.

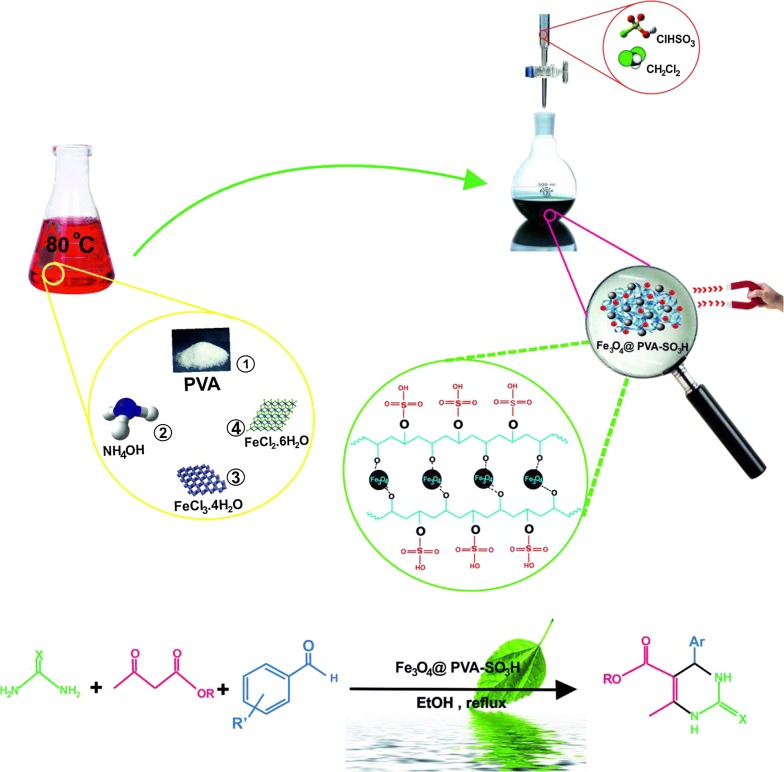

## Introduction

Recently, magnetic nanoparticles (MNPs) have raised awareness due to their potential application in catalytic activity [1, 2]. They have the advantage of both homogenous and heterogeneous catalyst including high reactivity, high dispersion and easy separation. These benefits are owning to their nanoscale size and magnetic properties [3–5]. Among all MNPs, Fe_3_O_4_ nanoparticles have received considerable amounts of researchers’ interests due to their low cost, majestic reactivity and high specific surface area which can be easily and rapidly isolated from the reaction mixture by using an external magnet [6]. Nowadays, the immobilization of biocompatible polymer onto magnetic nanoparticles have been highly taken into consideration by organic chemists [7–10].

Polyvinyl alcohol (PVA), a water-soluble synthetic biocompatible polymer has received great attentions due to its high hydrophilicity high density of –OH groups, low toxicity, low cost and high chemical resistance [11]. PVA was prepared from polyvinyl ester and has been applied widely in biomedical and industrial applications [12]. The large amount of OH groups and hydrophilicity nature of PVA are the major drawbacks of this synthetic polymer reducing its application. The main reason of this incident is dissolving in water. Noteworthy, hydrophilicity of PVA can be reduced via functionalizing OH groups [14].

Moreover, mechanical properties and water resistance can be improved by modifying PVA with chemical or physical cross-linkers. There are several reports about functionalizing OH with various groups such as acidic functional groups that can solve the hydrophilicity problem [13]. Over the past years, several methods have been announced for the synthesis Fe_3_O_4_/PVA nanocomposites such as electrospinning technique [15], ex situ [14] and in situ methods [16]. This synthesized nanocomposite has been utilized in various fields such as drug delivery as membranes for bone regeneration and other biomedical application [17, 18].

Proceeding our research on green nanocatalysts as well as multicomponent reaction (MCRs) [19–22] are considered as an important organic synthesis strategy. MCRs are one-pot reactions in which more than two reactants produce a single product that includes whole atoms of starting materials [23, 24]. Recently, MCRs have received a lot of attentions for producing various biologically active compounds. Dihydropyrimidinone (DHPM) derivatives are the most important class of heterocyclic compounds which have attracted lots of researcher’s attention due to their biochemical and pharmacological properties [25]. For the first time in 1891, Biginelli announced an useful reaction for the synthesis of DHPMs [26]. Because of the biological effects of DHPMs such as antiviral, antitumor, antibacterial and anti-inflammatory activities, several methods have been reported for synthesis of these compounds containing β-dicarbonyl compound, aldehyde and urea or thiourea in the presence of various catalysts such as Bronsted acid [27], Lewis acid [28], heteropolyacid [29] and Fe_3_O_4_ nanoparticles [30]. Most of these catalysts have several drawbacks such as tedious workup, toxic metals, low yields, long reaction time, environmental pollution and difficult separation. In the recent years, attempting to improve the catalyst in this reaction has received a lot of attention.

Herein, we report for the first time the synthesis and characterization of Fe_3_O_4_@PVA-SO_3_H nanocomposite film and investigate the catalytic application of this nanocomposite film synthesis of dihydropyrimidine (DHPM) derivatives.

## Experimental

### General

The solvents, chemicals, and reagents applied in our experiment were entirely purchased from Merck, Sigma and Aldrich. Melting points were measured on an Electrothermal 9100 apparatus and fourier transforms infrared spectroscopy (FT-IR) spectra were recorded through the method of KBr pellet on a Shimadzu IR-470 spectrometer. Adds that, ^1^H and ^13^C Nuclear Magnetic Resonance (NMR) spectra were done on a Bruker DRX-500 Avance spectrometer at 500 and 125 MHz, respectively. Scanning electron micrograph (SEM) images were also taken via Sigma-Zeiss microscope along with attached camera and transmission electron microscopy (TEM) was provided on a Philips CM200. To go through the details, magnetic measurements of the solid samples were performed using Lakeshore 7407 and Meghnatis Kavir Kashan Co., Iran vibrating sample magnetometers (VSMs). Elemental analysis of the nanocatalyst was carried out by energy-dispersive X-ray (EDX) analysis recorded Numerix DXP-X10P. XRD patterns of the solid powders were carried out using a JEOL JDX–8030 (30 kV, 20 mA). Nitrogen adsorption and desorption isotherms were determined using Micromeritics ASAP 2020 apparatus using nitrogen the analysis gas at − 196 °C. The specific surface areas were calculated by the BET method, and the pore size distributions were calculated from an adsorption branch of the isotherm by the BJH model. At final, we should add that the products were identified through the comparison between the spectroscopic/analytical data and those come from authentic samples.

### Preparation of Fe_3_O_4_@PVA nanocomposite film

To synthesize the Fe_3_O_4_@PVA nanocomposite film excellently, co-precipitation may consider the best approach. At first, a homogenous mixture resulted from 2.0 g of PVA 72,000 M_w_ constantly dissolved in 40 mL water (for 3 h at 80 °C). After that, under nitrogen (N_2_) atmosphere, homogenous PVA was mixed with 12 mL of NH_3_.H_2_O in a three-necked flask. Next step, 2.5 g of FeCl_3_·6H_2_O and 1.0 g of FeCl_2_·4H_2_O were dissolved in 10 mL of deionized water and the mixture was added slowly to the NH_3_-PVA solution. Then, in order to precipitate the Fe_3_O_4_@PVA, the mixture was heated for 120 min at 60 °C and washed with deionized water. At final, when the pH was hopefully reached to 7, the precipitation was dried at 80 °C in an oven.

### Preparation of Fe_3_O_4_@PVA-SO_3_H nanocomposite film

In the beginning, 0.5 g of Fe_3_O_4_@PVA in 20 mL CH_2_Cl_2_ was added to a suction flask equipped with a constant-pressure dropping funnel and a gas inlet tube which is conducting HCl gas over an adsorbing solution (i.e., water). While it dispersed by an ultrasonic bath for 30 min, a solution of chlorosulfonic acid (0.25 mL) in CH_2_Cl_2_ (5 mL) was supplemented dropwise at -10 °C. After that, in order to fetch up HCl totally, the mixture was at least stirred for 90 min. The consequence was hopefully a powder of nano-Fe_3_O_4_@PVA-SO_3_H was filtered and washed several times with dry CH_2_Cl_2_, methanol, and distilled water. The finalized nanocomposite was dried under vacuum at 70 °C.

### General procedure for the synthesis of DHPMs **4a**–**w**

0.05 g of Fe_3_O_4_@PVA-SO_3_H magnetic nanocatalyst was added into a solution consists of 1.50 mmol of an aromatic aldehyde, 1.50 mmol of a ß–ketoester, and 2.00 mmol of urea or thiourea. The mixture was timely refluxed in EtOH and the completion of the reaction was carefully monitored by thin layer chromatography (TLC). As a result, the catalyst was easily separated by an external magnet and the products were purely obtained from the recrystallization of the hot EtOH without more purification. Finally, we characterize some products through the FT-IR and some others via matching their melting points (Table 3) on literature samples.

### Spectral data of the selected products

#### Ethyl 4-(3-nitrophenyl)-6-methyl-2-oxo-1,2,3,4-tetrahydropyrimidine-5-carboxylate (**4c**):

^1^H NMR (500 MHz, CDCl_3_): δ_H_ (ppm) = 1.08 (3H, t, *J *= 7.1 Hz, CH_3_), 2.17 (3H, s, CH_3_), 3.93 (2H, q, *J *= 7.1 Hz, CH_2_), 6.11 (1H, d, *J *= 3.4 Hz, CH), 7.15–7.33 (5H, m, H–Ar), 7.74 (1H, s, NH), 9.19 (1H, s, NH); ^13^C NMR (125 MHz, CDCl_3_): δ_C_ (ppm) = 14.0, 15.9, 52.5, 60.7, 105.0, 121.5, 123.6, 127.5, 132.0, 132.5, 135.5, 140.6, 146.6, 160.6.

#### Ethyl 4-(4-hydroxyphenyl)-6-methyl-2-oxo-1,2,3,4-tetrahydropyrimidine-5-carboxylate (**4f**):

^1^H NMR (500 MHz, CDCl_3_): δ_H_ (ppm) = 1.06–1.09 (3H, t, *J *= 7 Hz, CH_3_), 2.21 (3H, s, CH_3_), 3.93–3.97 (2H, q, *J *= 6.5 Hz, CH_2_), 5.01 (1H, s, CH), 6.65–6.67 (2H, d, *J *= 8.5 Hz, H–Ar), 6.99–7.01 (2H, d, *J *= 8.5 Hz, H–Ar), 7.62 (1H, s, OH), 9.11 (1H, s, NH), 9.13 (1H, s, NH); ^13^C NMR (125 MHz, CDCl_3_): δ_C_ (ppm) = 14.5, 18.2, 53.8, 59.5, 100.0, 115.4, 127.8, 135.8, 148.2, 152.6, 156.9, 165.8.

#### Ethyl 4-(4-fluorophenyl)-6-methyl-2-oxo-1,2,3,4-tetrahydropyrimidine-5-carboxylate (**4j**):

^1^H NMR (500 MHz, CDCl_3_): δ_H_ (ppm) = 1.05 (3H, CH_3_), 2.22 (3H, s, CH_3_), 3.94 (2H, q, CH_2_), 5.12 (1H, s, CH), 7.16 (2H, H–Ar), 7.22 (2H, H–Ar), 7.75 (1H, s, NH), 9.23 (1H, s, NH); ^13^C NMR (125 MHz, CDCl_3_): δ_C_ (ppm) = 14.5, 18.2, 53.7, 59.6, 99.5, 115.5, 115.6, 128.7, 141.5, 149.0, 152.4, 160.7, 162.7, 165.6.

#### Ethyl 4-(3-hydroxyphenyl)-6-methyl-2-thioxo-1,2,3,4-tetrahydropyrimidine-5-carboxylate (**4r**):

^1^H NMR (500 MHz, CDCl_3_): δ_H_ (ppm) = 1.07–1.123 (3H, t, *J *= 11.5 Hz, CH_3_), 3.45 (3H, s, CH_3_), 3.95–4.00 (2H, q, *J *= 11.5 Hz, CH_2_), 5.05 (1H, s, CH), 6.65–6.69 (2H, d, *J *= 8.5 Hz, H–Ar), 7.55–7. 153 (2H, d, *J *= 8.5 Hz, H–Ar), 9.45 (1H, s, NH), 9.11 (1H, s, NH), 9.13 (1H, s, OH).

#### Methyl 6-methyl-2-oxo-4-phenyl-1,2,3,4-tetrahydropyrimidine-5-carboxylate (**4s**):

^1^H NMR (500 MHz, DMSO): δ_H_ (ppm) = 2.21 (3H, s, CH_3_), 3.49 (3H, s, CH_3_), 5.10 (1H, d, *J *= 3.3 Hz, CH), 7.18–7.29 (5H, m, H–Ar), 7.72 (1H, s, NH), 9.18 (1H, s, NH); ^13^C NMR (125 MHz, CDCl_3_); δ_C_ (ppm) = 18.7, 51.3, 55.6, 101.2, 126.6, 128.1, 128.9, 143.7, 146.9, 153.9, 166.3.

#### Methyl 4-(4-chlorophenyl)-6-methyl-2-oxo-1,2,3,4-tetrahydropyrimidine-5-carboxylate (**4t**):

^1^H NMR (500 MHz, CDCl_3_): δ_H_ (ppm) = 2.31 (3H, s, CH_3_), 3.59 (3H, s, CH_3_), 5.26 (1H, d, *J *= 3.5 Hz, CH), 7.26 (4H, m, H–Ar), 7.51 (1H, s, NH), 9.11 (1H, s, NH); ^13^C NMR (125 MHz, CDCl_3_); δ_C_ (ppm) = 18.7, 52.6, 57.7, 98.9, 121.2, 123.6, 127.5, 135.0, 142.6, 146.6, 152.6.

#### Methyl 4-(3-hydroxyphenyl)-6-methyl-2-oxo-1,2,3,4-tetrahydropyrimidine-5-carboxylate (**4v**):

^1^H NMR (500 MHz, CDCl_3_): δ_H_ (ppm) = 2.22 (3H, s, CH_3_), 3.52 (3H, s, CH_3_), 5.04 (1H, s, CH), 6.59–6.65 (3H, m, H–Ar), 7.03 (1H, m, H–Ar), 7.08 (1H, s, OH), 9.22 (1H, s, NH), 9.38 (1H, s, NH); ^13^C NMR (125 MHz, CDCl_3_); δ_C_ (ppm) = 18.3, 51.3, 54.1, 99.5, 113.4, 114.6, 117.2, 129.8, 146.5, 148.9, 152.8. 157.8, 166.3.

## Results and discussion

**Scheme 1:**
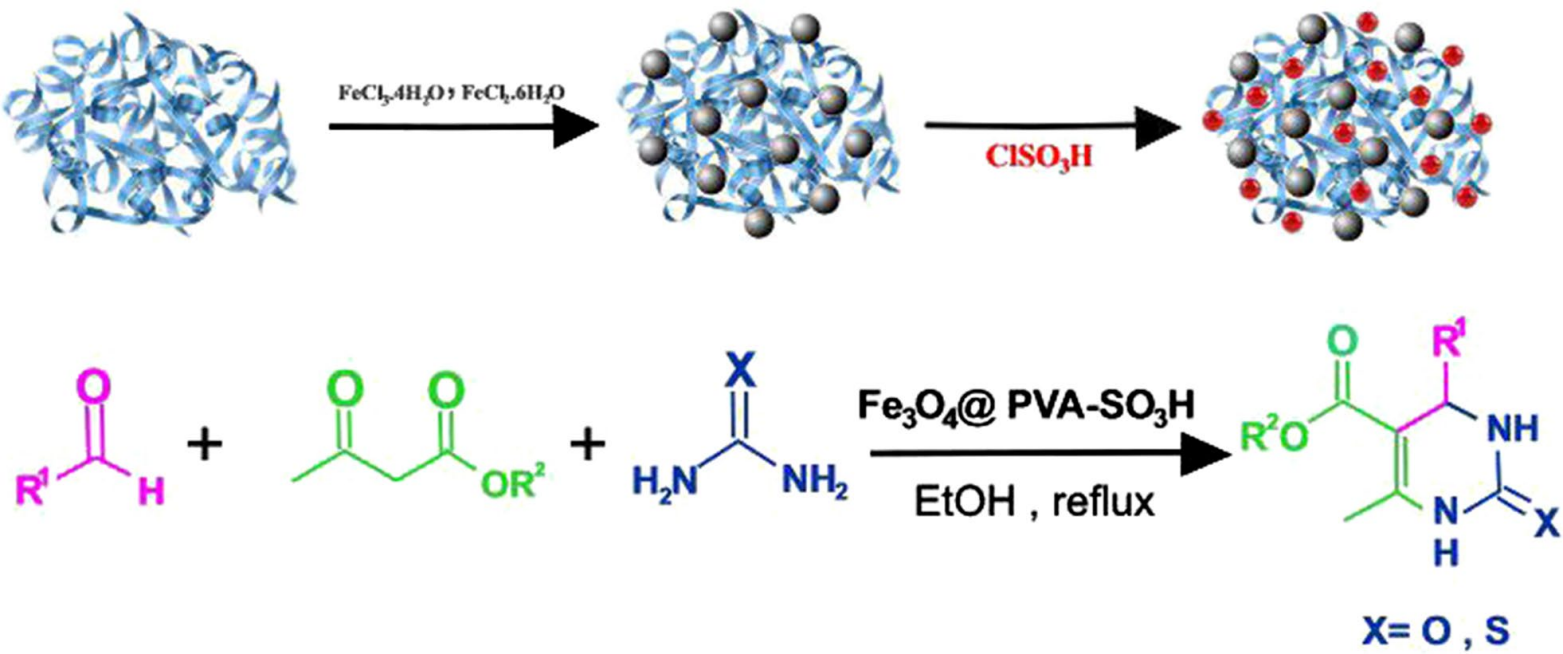
(a) Preparation of: Fe_3_O_4_@PVA-SO_3_H and (b) the synthesis of DHPMs **4a**–**w** in the presence of Fe_3_O_4_@PVA-SO_3_H

In this work, Fe_3_O_4_@PVA-SO_3_H magnetic nanocatalyst was synthesized after two steps under mild conditions. As it is illustrated in Scheme 1, according to the co-precipitation method, the Fe_3_O_4_@PVA nanoparticles were synthesized under N_2_ and in presence of PVA, solution of FeCl_3_.6H_2_O and FeCl_2_.4H_2_O. Then, in order to achieve Fe_3_O_4_@PVA-SO_3_H nanocatalyst, Fe_3_O_4_@PVA was reacted by chlorosulfonic acid and analyzed by several methods. At final, the nanocomposite successfully applied as an effective catalyst in the synthesis of DHPM derivatives.

### Characterization of the nanocomposite

#### FT-IR analysis

**Fig. 1:**
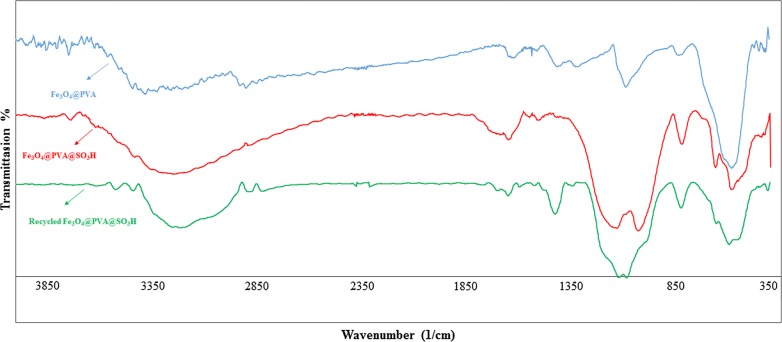
The FT-IR spectra of: Fe_3_O_4_@PVA, Fe_3_O_4_@PVA-SO_3_H and recycled Fe_3_O_4_@PVA-SO_3_H

To study the interactions of PVA film and Fe_3_O_4_ nanoparticles, FT-IR analysis may consider one of the best tools. As can be seen in Fig. 1, the broad band in 3015–3529 cm^−1^ obviously stems from the vibration of OH, hydrogen bonds of OH groups in PVA and absorbed moisture. Another strong band in 2908–2920 cm^−1^ also indicates that there is an asymmetric stretch vibration in C–H groups. Moreover, the peaks on 1443–1460 cm^−1^ and 1500–1250 cm^−1^, respectively refer to the C–H bending of CH_2_ and the tensile vibration of C=O or C–O–C in the PVA spine. In other words, Fe_3_O_4_ nanoparticles may interact with PVA via hydroxyl groups present on their surfaces. On the other hand, the presence of iron oxide in the hydrogel is aligned by the absorption bands in 480–500 cm^−1^. Thus, the peaks in 400–600 cm^−1^ may demonstrate the deformation of the iron oxide structure and the OH groups on the surface of the Fe_3_O_4_ nanoparticles. The vibration band of Fe–O–C bond in 1000–1100 cm^−1^ also confirms the interactions between PVA and Fe_3_O_4_ nanoparticles.

#### Energy-dispersive X-ray (EDX)

**Fig. 2:**
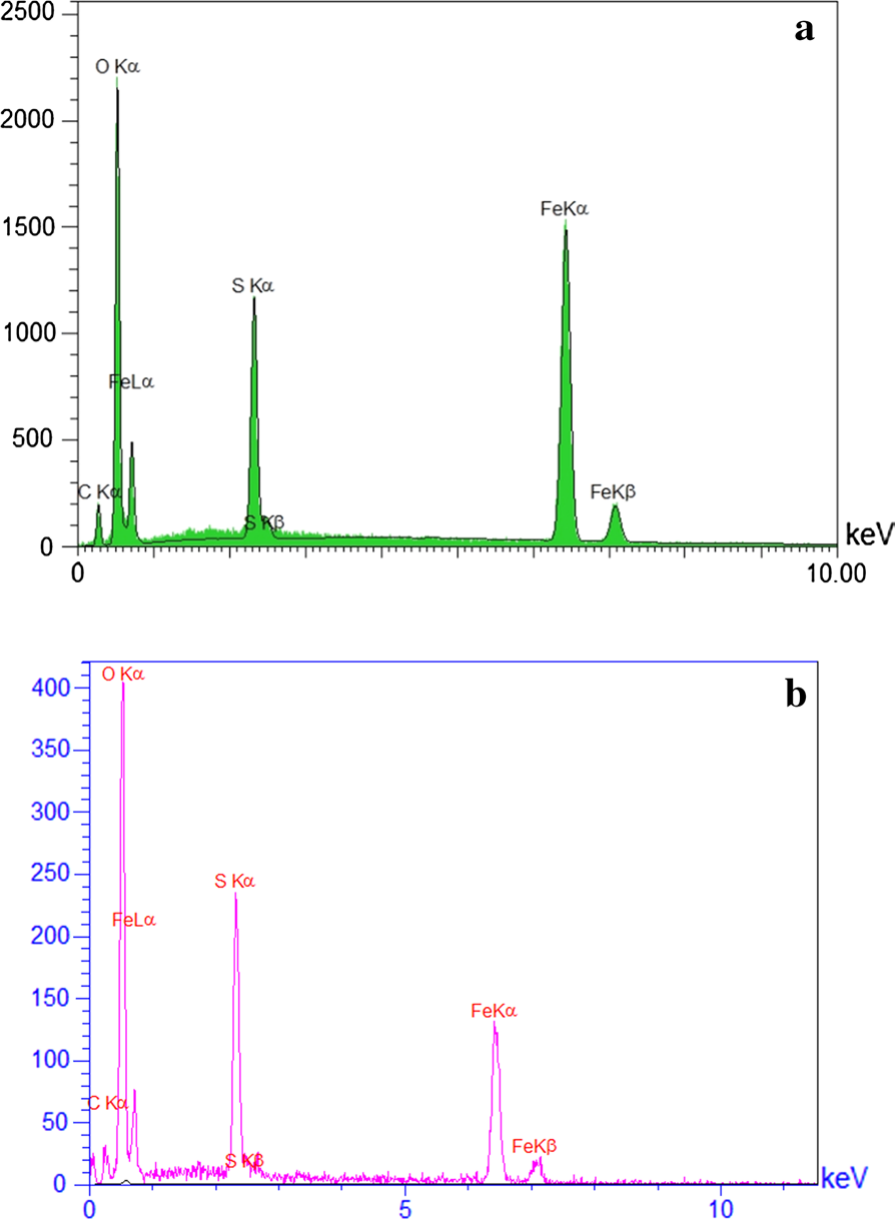
EDX analysis of: **a** fresh Fe_3_O_4_@PVA-SO_3_H and **b** the recycled Fe_3_O_4_@PVA-SO_3_H

EDX analysis (Fig. 2a) was included to investigate the polymer film and the well-sulfonated process in Fe_3_O_4_ nanoparticles. In this way, although the exact ratio of Fe^2 +^/Fe^3 +^ might not be obtained through the EDX analysis, there are two groups of peaks who may have the significant information. First, the peaks in 0.75, 6.5 and 7.1 possibly characterize the presence of Fe atoms and second, the peaks in 0.5, 0.25, represent the O and C elements in PVA. Briefly, not only do these peaks lucidly show that the sample mainly includes PVA, Fe_3_O_4_ and SO_3_H, but also there is not any kind of impurity according to the EDX chart. Figure 2b confirmed that there is no considerable difference between the values ​​of the elements in primary catalyst and recycled catalyst.

#### Scanning electron microscopy (SEM)

**Fig. 3:**
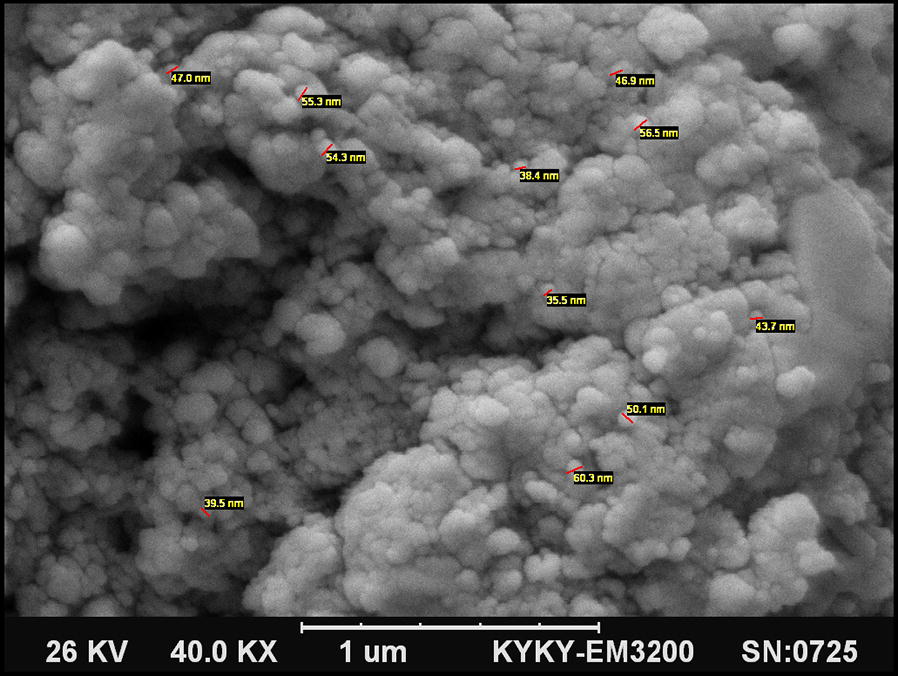
The SEM image of Fe_3_O_4_@PVA-SO_3_H nanocomposite film

As a matter of fact, the elaborations related to the morphology and size of the nanocatalyst must be also explored. Therefore, we adopt SEM to investigate the morphology of the pure PVA and prepared nanocomposite. As it is shown in Fig. 3, the roughness may refer to the presence of Fe_3_O_4_ particles amongst the PVA matrix. Furthermore, not only is there not any Fe_3_O_4_ aggregation, but also the nanocomposite particles are distributed uniformly in an average size of 47 nm. It is worth noting that the Fe_3_O_4_ particles have the nearly spherical shape and are part of the Fe_3_O_4_@PVA-SO_3_H nanocomposite film. On the other hand, because there is an appreciable adhesion between organic (PVA) and inorganic (Fe_3_O_4_) phase, the distance between the nanoparticles is much larger than diameter of them.

#### Transmission electron microscopy (TEM)

**Fig. 4:**
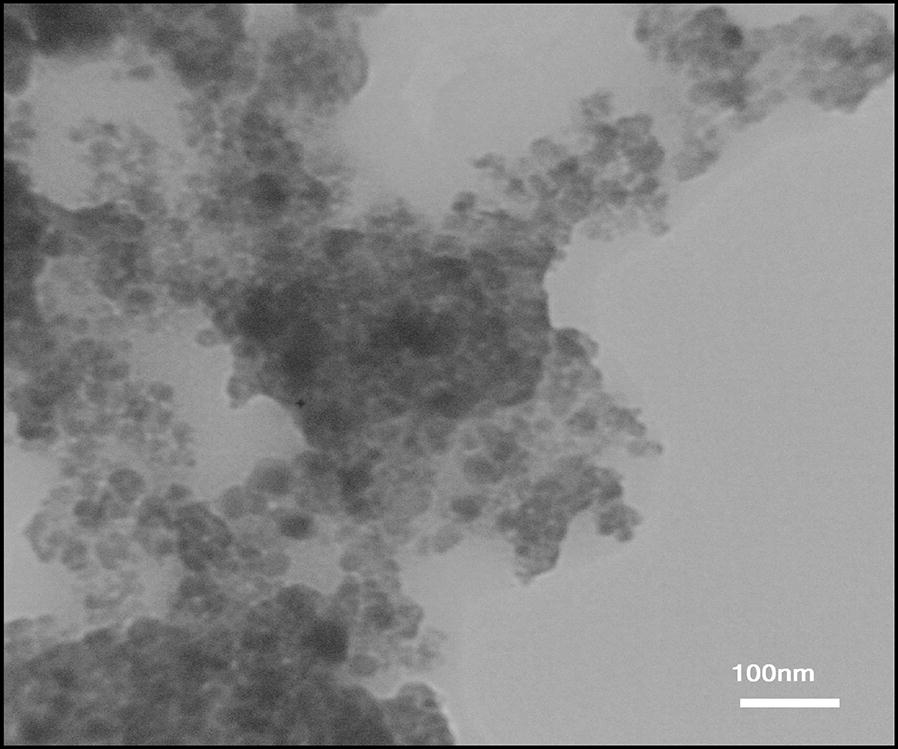
The TEM image of Fe_3_O_4_@PVA-SO_3_H nanocomposite film

To lend further support the morphology of the synthesized catalyst, we also include the TEM images in our study. In Fig. 4, the magnetic nanoparticles are shown by dark spots. Some of them who are marked more solid seem to be severely agglomerated. However, most they are not. In contrast, polyvinyl alcohol might be recognized by transparent color in the TEM images. Amazingly, the spherical magnetic nanoparticles who are homogenously distributed prove that polyvinyl alcohol successfully prevent of coagulation.

#### Thermogravimetric analysis (TGA)

**Fig. 5:**
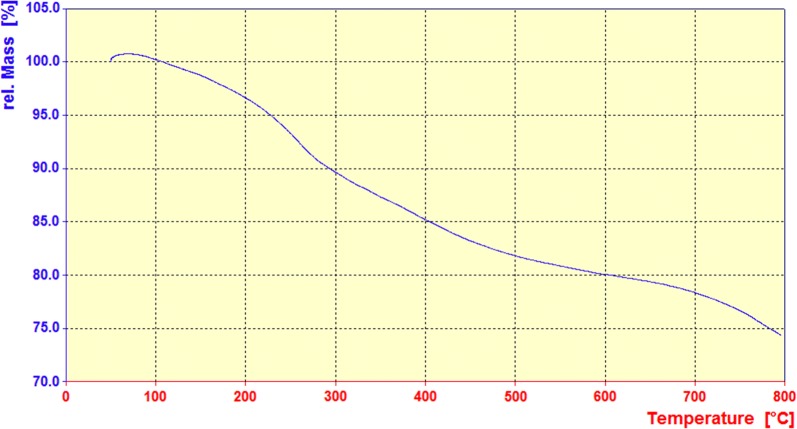
The TGA curve of Fe_3_O_4_@PVA-SO_3_H nanocomposite film

The thermal behaviour of the prepared Fe_3_O_4_*@*PVA-SO_3_H magnetic nanocomposite film was investigated by thermo gravimetric analysis (TGA) over the temperature range of 20–800 °C under air atmosphere. According to the TG curve of MGCS in Fig. 5, the first weight loss (from 50 to 150 °C) denotes the evaporation of adsorbed water in the sample. The second weight loss (from 200 to 550 °C) occurs when the PVA and SO_3_H groups are decomposed. And, up to 270 °C, there is not any weight loss in the nanocomposite (it is stable at least until 250 °C). In conclusion, this synthesized film is suitable for organic reactions outright because it has a higher thermal stability in comparison with PVA.

#### X-ray diffraction (XRD)

**Fig. 6:**
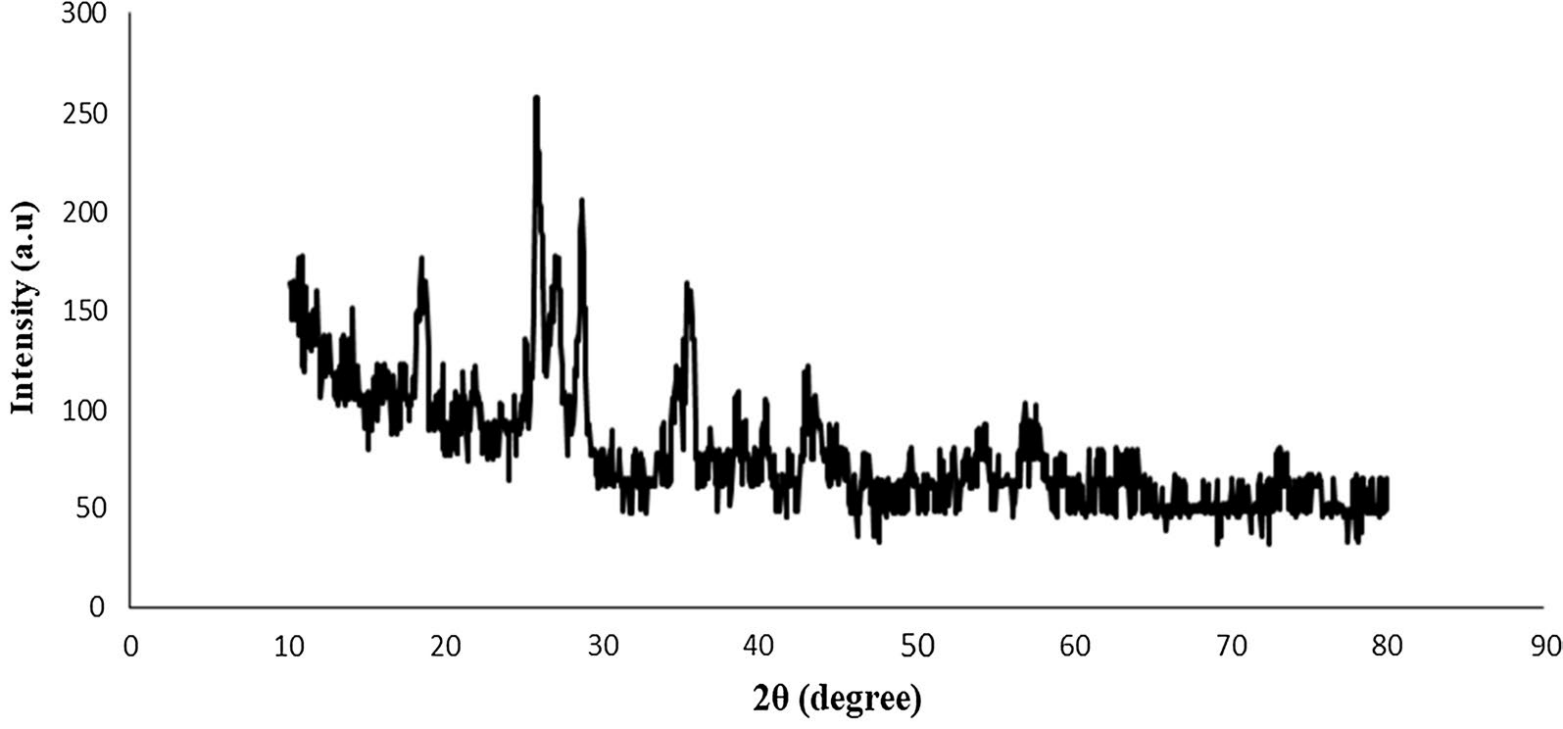
XRD pattern of Fe_3_O_4_@PVA-SO_3_H nanocomposite film

XRD may be opted by any scientist who would like to study the crystallographic structure of the nanocomposites. In fact, the structure and phase are be able to qualitatively recognize, if one study angles and relative intensity of the peaks within the XRD analysis. Amorphous materials are definitely without peaks. However, crystalline ones who are established organized structure show specific angles in XRD. The XRD pattern of the Fe_3_O_4_@PVA-SO_3_H nanocomposite is shown in Fig. 6 and the average size of the particles is calculated by the Scherrer equation; D = kλ/β cosθ. According to the figure, there is a large reflection at 2θ = 19.4° for the PVA film. However, based on the Fig. 6, the diffraction peaks at the dispersion angle (2θ) are 30.39, 35.81, 37.46, 54.01, 57.58, 63.25, 66.51, 74.86 and 75.88. So, there are strong correlations between the pattern and standard JCPDS Card No. (01-075-0449) and the decrease in the intensity of the pixels fairly declines the interaction between poly(vinyl) alkyl and iron oxide nanoparticles (the crystallization).

#### Vibrating sample magnetometer (VSM)

**Fig. 7:**
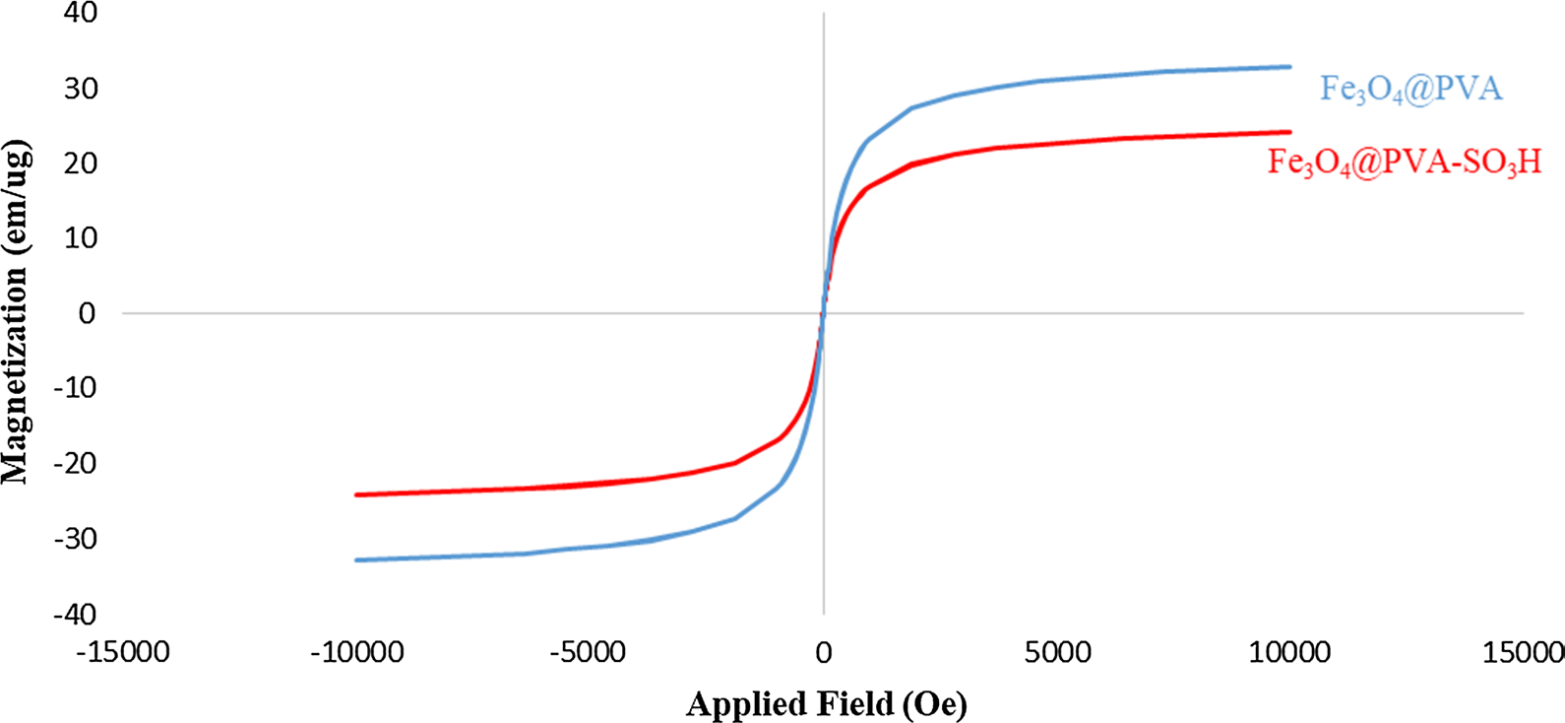
VSM of Fe_3_O_4_@PVA and Fe_3_O_4_@PVA-SO_3_H nanocomposite film

VSM analysis was applied at room temperature to measure magnetic properties. M and H curves are illustrated in Fig. 7 for Fe_3_O_4_@PVA and Fe_3_O_4_@PVA-SO_3_H composite nanoparticles, respectively. Both of them show a phenomenal paramagnetic behaviour without any obstruction or inclination. In fact, in the range of applied field with intensity of 10 kOe, for both the maximum magnetic saturation (Ms) is 32.95 emu/g and 24.15 emu/g, respectively. The amount of saturation absorption may be attributed to the SO_3_H which is coated on the nanocomposite and eliminates the accumulation and formation of the large clusters. This results in the decrease in the size of the crystal and the amount of Ms.

#### Brunauer–Emmett–Teller (BET)

**Fig. 8:**
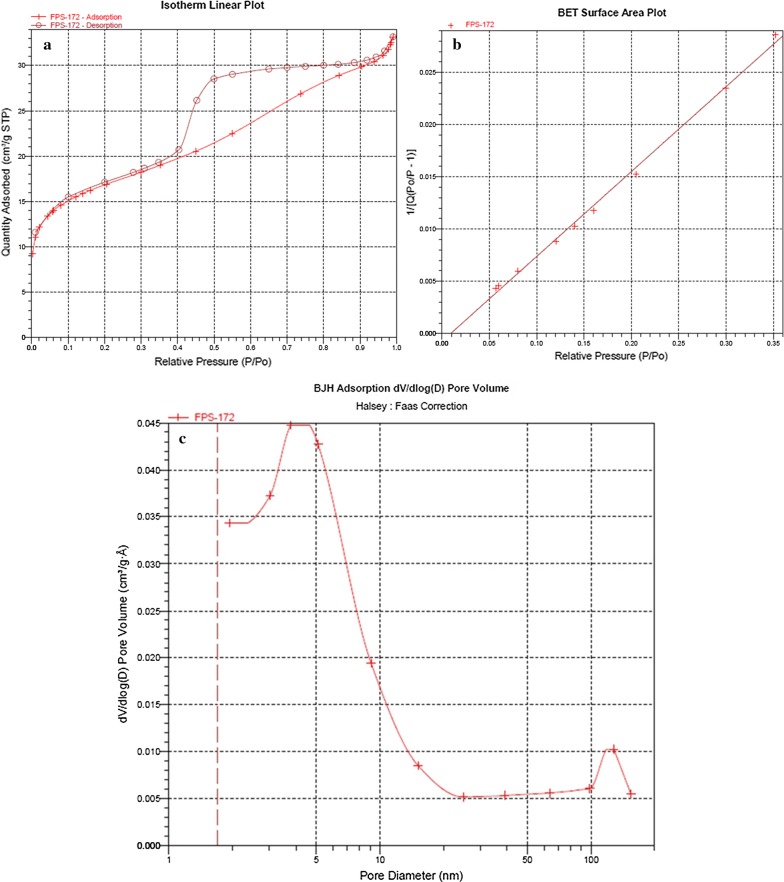
N_2_ adsorption–desorption isotherm of: **a** isotherm linear plot, **b** BET surface area plot and **c** BJH adsorption of pore-size distribution curve of Fe_3_O_4_@PVA-SO_3_H

The N_2_ adsorption/desorption isotherm of Fe_3_O_4_@PVA@SO_3_H composite is shown in Fig. 8, which displays a typical type IV curve, indicating the presence of mesoporous structure. The BET surface area, BJH pore volume and pore size is 54.052 m^2^/g, 0.042 cm^3^/g, and 3.48 nm, respectively. These results confirms relatively suitable specific surface area maintenance within the nanocomposite preparation and functionalization of MNPs.

#### Back titration of Fe_3_O_4_@PVA-SO_3_H in aqueous media

Acidity ([H+]) of the synthesized Fe_3_O_4_@PVA-SO_3_H nanocatalyst was explored by the back titration method. At first, 0.5 g of Fe_3_O_4_@PVA-SO_3_H, 0.5 g of NaCl, and 10 mL of NaOH 0.1 M were added to 35 mL of distilled water and stirred with a magnet for 24 h. After that, a few drops of phenolphthalein were supplemented into the mixture and the colour changed to pink. Finally, the mixture was titrated by the solution of HCl 0.1 M to reach the neutral pH. Accordingly, the pH of the nanocatalyst was calculated 1.61.

### Catalytic application of Fe_3_O_4_@PVA-SO_3_H in the synthesis of DHPMs

In order to look into the catalytic activity of the nanocatalyst, we apply a one-pot synthesis of DHPMs derivatives. At first, the reaction conditions is optimized through the condensation of 1.5 mmol of ethyl acetoacetate **1**, 1.5 mmol of benzaldehyde **2** and 2 mmol of urea **3** in the presence of different catalytic amounts of Fe_3_O_4_@PVA-SO_3_H in EtOH and under reflux conditions. Table 1 represents that 0.01 g of catalyst was enough to catalyze the reactions produce high yields of DHPMs derivatives. On the other side, the efficiency and the yield of the reaction model in EtOH were meaningfully higher than those in other solvents and in short reaction times (Table 2). Furthermore, we made a considerable comparison between our catalysts and several others who were previously reported and widely adopted to synthesize DHPMs derivatives. Table 3 greatly summarizes them and proposes that our work is hugely in favor of the saving energy, high yields of the products and the reusability of the nanocatalyst.

**Table 1:** Optimization of reaction conditions using different catalytic amounts

Entry	Solvent	Catalyst	Amount (mg)	Time (min)	Yield^a^ (%)
1	EtOH	–	–	10	Trace
2	EtOH	Fe_3_O_4_@PVA-SO_3_H	10	10	65
3	EtOH	Fe_3_O_4_@PVA-SO_3_H	30	10	82
4	EtOH	Fe_3_O_4_@PVA-SO_3_H	40	10	95
5	EtOH	Fe_3_O_4_@PVA-SO_3_H	50	10	99
6	EtOH	Fe_3_O_4_@PVA-SO_3_H	60	10	99
7	EtOH	Fe_3_O_4_@PVA-SO_3_H	70	10	99

^a^Isolated yield

**Table 2:** Optimization of reaction conditions using various solvents

Entry	Solvent	Catalyst	Time (min)	Conditions	Yield^a^ (%)
1	EtOH	–	–	Reflux	Trace
2	EtOH	Fe_3_O_4_@PVA	50	Reflux	Trace
3	EtOH	Fe_3_O_4_@PVA-SO_3_H	10	Reflux	99
4	EtOH	Fe_3_O_4_@PVA-SO_3_H	20	r.t.	70
5	MeOH	Fe_3_O_4_@PVA-SO_3_H	10	Reflux	90
6	H_2_O	Fe_3_O_4_@PVA-SO_3_H	20	Reflux	65
7	CH_3_CN	Fe_3_O_4_@PVA-SO_3_H	10	Reflux	85
8	PEG-400	Fe_3_O_4_@PVA-SO_3_H	20	Reflux	95
9	CH_2_Cl_2_	Fe_3_O_4_@PVA-SO_3_H	20	Reflux	68

^a^ Isolated yield

**Table 3:** Comparison of the efficiency of Fe_3_O_4_@PVA-SO_3_H with that of other reported catalysts in the synthesis of model **4a**

Entry	Catalyst	Conditions	Time	Yield (%)	Ref
1	SnCl_2_/nano SiO_2_	EtOH/reflux	40 min	94	[31]
2	Silica-bonded N-propyl sulfamic acid (SBNPSA)	EtOH/reflux	3–4 h	90–95	[32]
3	nanoZnO (5 mol %)	Solvent free/60 °C	10 h	95	[33]
4	NH_4_H_2_PO_4_ (5 mol %) or NH_4_H_2_PO_4_/SiO_2_	Solvent free/100 °C	2 h	85	[34]
5	Fe_3_O_4_@mesoporous SBA-15	EtOH/65 °C	6 h	85	[35]
6	Fe_3_O_4_@PVA-SO_3_H (50 mg)	EtOH/reflux	10 min	99	This work

^a^Isolated yield

It should be add that our strategy is be able to powerfully apply to a very wide range of synthesises. For instance, a broad range of aromatic aldehydes possessing electron-withdrawing and electron-releasing substitutions, were employed and as a result a different array of products were synthesized in an appropriate time. Table 4 contains all the aromatic aldehydes supplied the desired products with high-to-excellent yields and in short reaction times.

**Table 4:** Synthesis of DHPMs **4a**–**w** by using Fe_3_O_4_@PVA-SO_3_H under refluxing conditions

Entry	R^1^	R^2^	X	Product	Time (min)	Yield^a^ (%)	Mp (°C)	
							Found	Reported
1	C_6_H_5_	Et	O	**4a**	10	99	201–202	201 [19]
2	4-ClC_6_H_4_	Et	O	**4b**	10	98	210–212	213 [36]
3	3-O_2_NC_6_H_4_	Et	O	**4c**	10	97	225–226	224–226 [36]
4	4-O_2_NC_6_H_4_	Et	O	**4d**	10	98	208–209	206–208 [36]
5	2,4-(Cl)_2_C_6_H_3_	Et	O	**4e**	10	95	248–250	248–250 [32]
6	4-OHC_6_H_4_	Et	O	**4f**	12	90	230–231	231–233 [36]
7	3,4,5-(CH_3_O)_3_C_6_H_2_	Et	O	**4g**	10	87	178–180	178–180 [37]
8	3-OHC_6_H_4_	Et	O	**4h**	15	85	222–223	221 [19]
9	3,4-(OH)_2_C_6_H_3_	Et	O	**4i**	20	80	247–248	243–244 [38]
10	4-FC_6_H_4_	Et	O	**4j**	10	99	181–182	181–183 [39]
11	4-BrC_6_H_4_	Et	O	**4k**	10	98	215–217	213 [19]
12	2-OHC_6_H_4_	Et	O	**4l**	15	92	201–203	198–200 [37]
13	2-Thienyl	Et	O	**4m**	15	95	203–204	200–202 [40]
14	2-Pyridyl	Et	O	**4n**	15	95	181–183	182–184 [40]
15	2-Furanyl	Et	O	**4o**	10	95	212–213	211–213 [40]
16	C_6_H_5_	Et	S	**4p**	10	98	204–205	203 [19]
17	4-FC_6_H_4_	Et	S	**4q**	10	96	180–181	179–181 [39]
18	3-OHC_6_H_4_	Et	S	**4r**	20	82	184–186	184–186 [37]
19	C_6_H_4_	Me	O	**4s**	10	98	215–218	215–218 [36]
20	4-ClC_6_H_4_	Me	O	**4t**	10	97	204–206	205–207 [36]
21	4-MeC_6_H_4_	Et	O	**4u**	10	92	208–210	209–210 [36]
22	3-OHC_6_H_4_	Me	O	**4v**	15	84	224–225	222 [19]
23	C_6_H_4_	Me	S	**4w**	10	97	224–227	222–224 [36]

^a^Isolated yield

### Mechanism evaluation

**Scheme 2:**
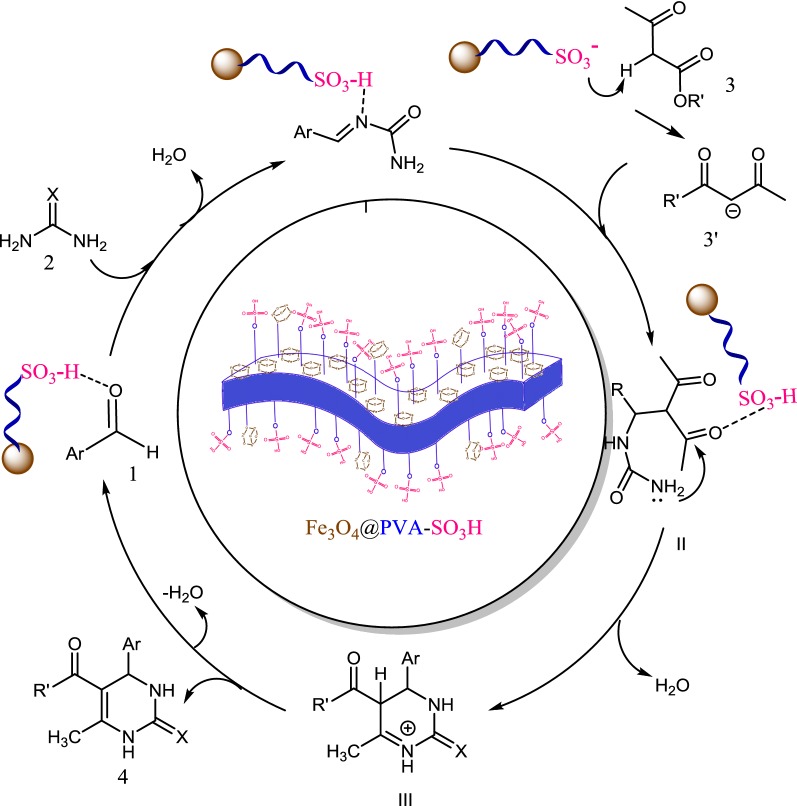
Plausible mechanism for the synthesis of DHPM derivatives by Fe_3_O_4_@PVA-SO_3_H magnetic nanocatalyst

Scheme 2 suggests a mechanism for the synthesis of DHPMs derivatives. Initially, intermediate **I** is formed by reaction of the aldehyde with urea or thiourea in the presence of Fe_3_O_4_@PVA-SO_3_H. Subsequently, the addition of the ß-ketoester is followed by cyclization and dehydration, and finally dihydropyrimidinone is synthesized.

### Reusability of Fe_3_O_4_@PVA-SO_3_H magnetic nanocatalyst

**Fig. 9:**
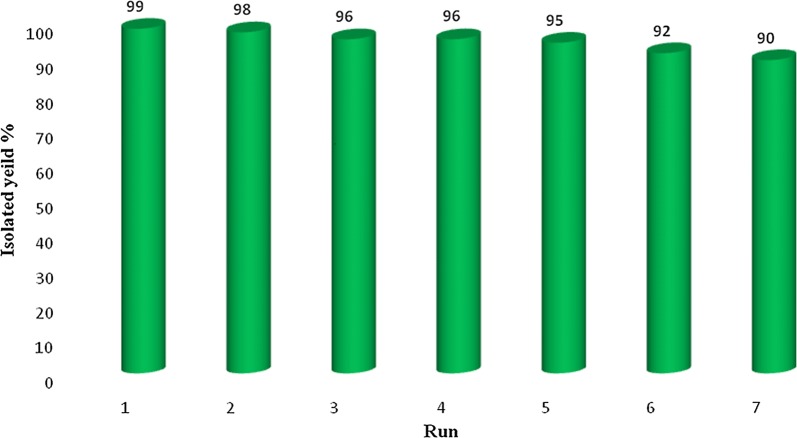
Recycling diagram of Fe_3_O_4_@PVA-SO_3_H nanocatalyst in the synthesis of **4a**

The reusability perhaps is one of the most substantial advantages the catalysts may have and it play the key role in commercial applications. For that matter, the reusability of Fe_3_O_4_@PVA-SO_3_H nanocatalyst was also studied in the reaction model. In this way, after completion of the reaction, the nanocatalyst were separated by an external magnet, washed with ethanol, dried and lastly reused in subsequent reactions. Surprisingly, the nanocatalyst could be reused at least six times without any appreciable loss of the yields in products (Fig. 9).

## Conclusions

In summary, we have introduced Fe_3_O_4_@PVA-SO_3_H nanocomposite film prepared by a facile one-step in situ green precipitation method. FT-IR, EDX, VSM, TGA, XRD, SEM and TEM were applied to confirm the formation of nanocomposite. FT-IR spectrum confirmed the presence of Fe–O of Fe_3_O_4_, PVA hydroxyl and S=O bonds of sulfonated groups, indicating the formation of the nanocomposite. EDX analysis showed the presence of C, S, O and Fe elements. In XRD pattern, the expected peaks were observed in accordance with standard cards of Fe_3_O_4_ MNPs and PVA film. TEM images indicated the uniform dispersion of nanoparticles in the PVA polymer matrix, as well as polyvinyl alcohol prevented the agglomeration of MNPs. It has been proven by SEM images that spherical Fe_3_O_4_ particles are distributed uniformly in a medium size of 47 nm in the PVA films. The VSM curve shows that with the sulfonation of the Fe_3_O_4_@PVA nanocatalyst, only 8.8 emu/g of magnetic property has been reduced, which indicates the presence of functional groups in the nanocomposite. TGA results exhibited that the nanocomposite was stable at least until 250 °C without considerable mass loss. The BET-BJH showed reasonable data for surface area, pore volume and pore size of 54.052 m^2^/g, 0.042 cm^3^/g and 3.48 nm, respectively. This magnetic nanocomposite film was applied as a catalyst for the synthesis of DHPM derivatives. The catalyst can be easily separated by an external magnet and recycled for six times without any appreciable loss of activity. Some of the advantageous of the present protocol are reusability of the catalyst high-to-excellent yields, mild reaction conditions and easy work up procedure. Furthermore, FT-IR, ^1^H and ^13^C NMR analyses were performed for the confirmation of the synthesized organic products, DHPMs. Finally, this is the first report on design, synthesis, functionalization and characterization of the present nanocomposite film and performance as a heterogeneous catalyst in organic reactions.

## Additional file

**Additional file 1.** Supporting information.
