# Supplementary material for: Design and preparation of Fe3O4@PVA polymeric magnetic nanocomposite film and surface coating by sulfonic acid via in situ methods and evaluation of its catalytic performance in the synthesis of dihydropyrimidines
Source: BMC Chem. 2019 Feb 4;13(1):19. doi: 10.1186/s13065-019-0538-2 (PMC6661808; doi:10.1186/s13065-019-0538-2)
Supplement: Supplementary file 1 — Additional file 1. Supporting information. [file 13065_2019_538_MOESM1_ESM.pdf]

Supplementary Information

Design and preparation of Fe_3O_4@PVA polymeric magnetic nanocomposite film and surface coating by sulfonic acid via in-situ methods and evaluation of its catalytic performance in the synthesis of dihydropyrimidines

Ali Maleki*, Maryam Niksefat, Jamal Rahimi, Zoleikha Hajizadeh

Catalysts and Organic Synthesis Research Laboratory, Department of Chemistry, Iran University of Science and Technology, Tehran 16846-13114, Iran

**Corresponding author. E-mail: maleki@iust.ac.ir; Fax: +98-21-73021584; Tel: +98-21-77240540-50*

Table of contents

Subject *Page*

Figure S1. ^{1}H NMR spectrum of the product **4c** S2
Figure S2. ^{13}C NMR spectrum of the product **4c** S3
Figure S3. ^{1}H NMR spectrum of the product **4f** S4
Figure S4. ^{13}C NMR spectrum of the product **4f** S5
Figure S5. ^{1}H NMR spectrum of the product **4j** S6
Figure S6. ^{13}C NMR spectrum of the product **4j** S7
Figure S7. ^{1}H NMR spectrum of the product **4r** S8
Figure S8. ^{1}H NMR spectrum of the product **4s** S9
Figure S9. ^{13}C NMR spectrum of the product **4s** S10
Figure S10. ^{1}H NMR spectrum of the product **4t**......S11
Figure S11. ^{13}C NMR spectrum of the product **4t** S12
Figure S12. ^{1}H NMR spectrum of the product **4v** S13
Figure S13. ^{13}C NMR spectrum of the product **4v**......S14

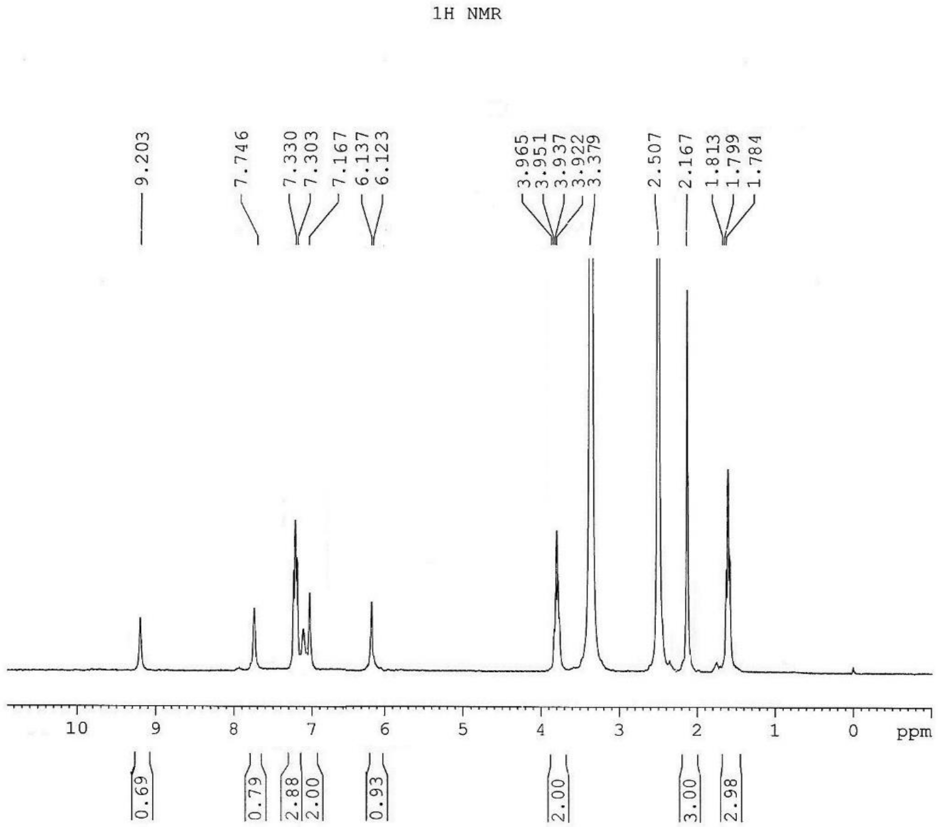

Figure S1. ^{1}H NMR spectrum of the product **4c**.

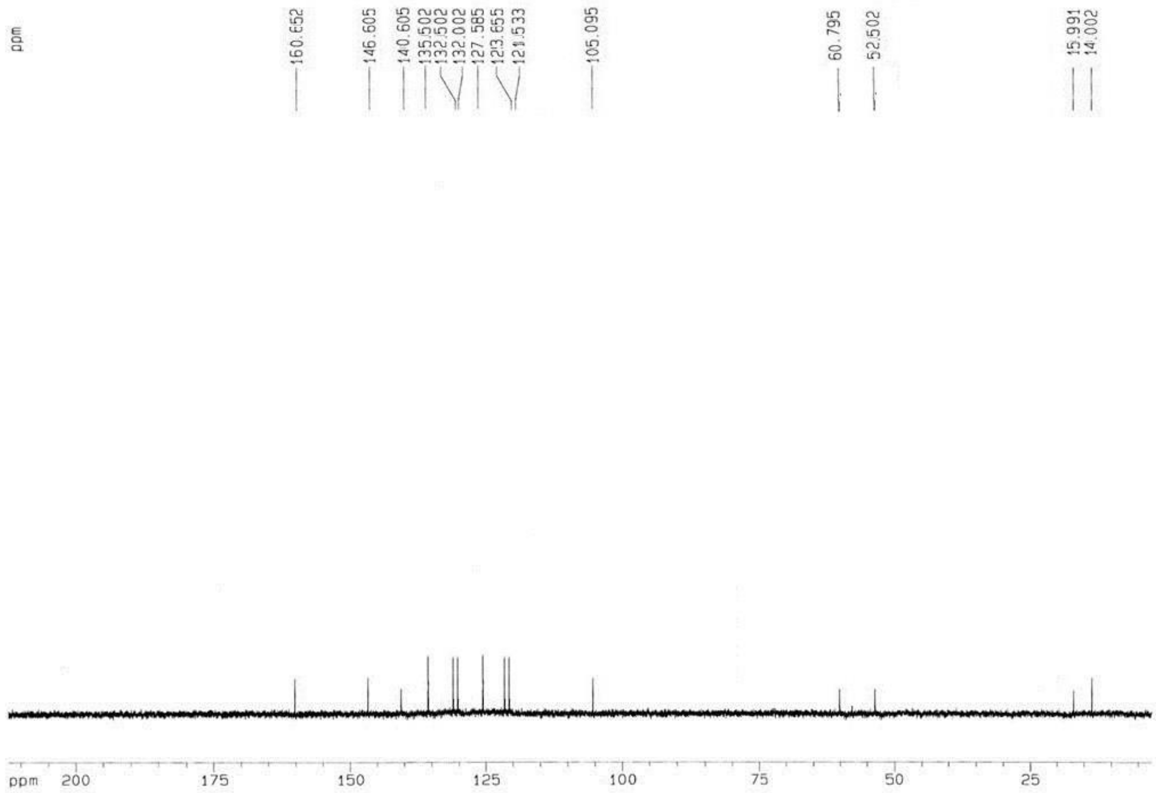

Figure S2. ^{13}C NMR spectrum of the product **4c**.

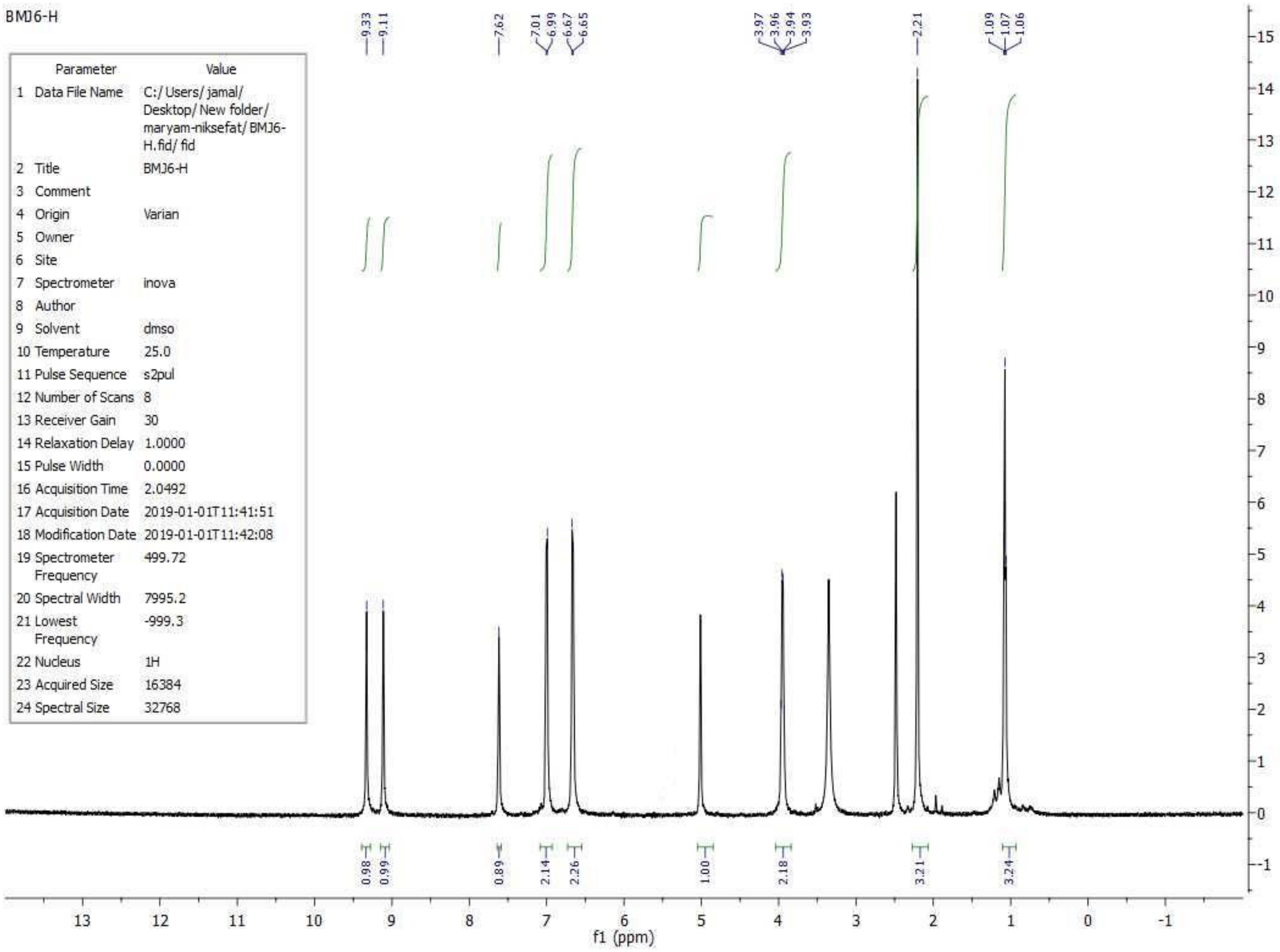

Figure S3. ^{1}H NMR spectrum of the product **4f**.

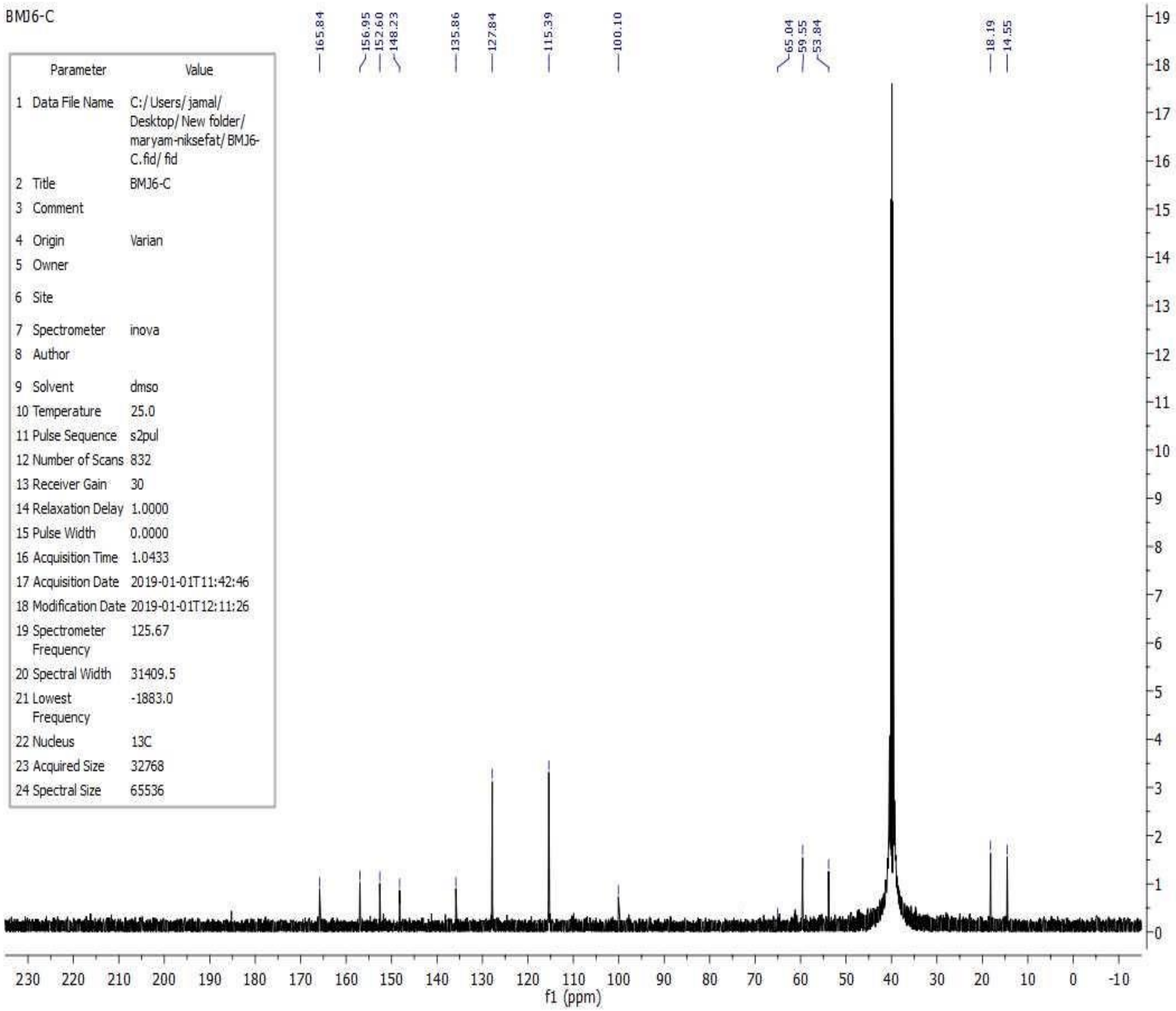

Figure S4. ^{13}C NMR spectrum of the product **4f.**

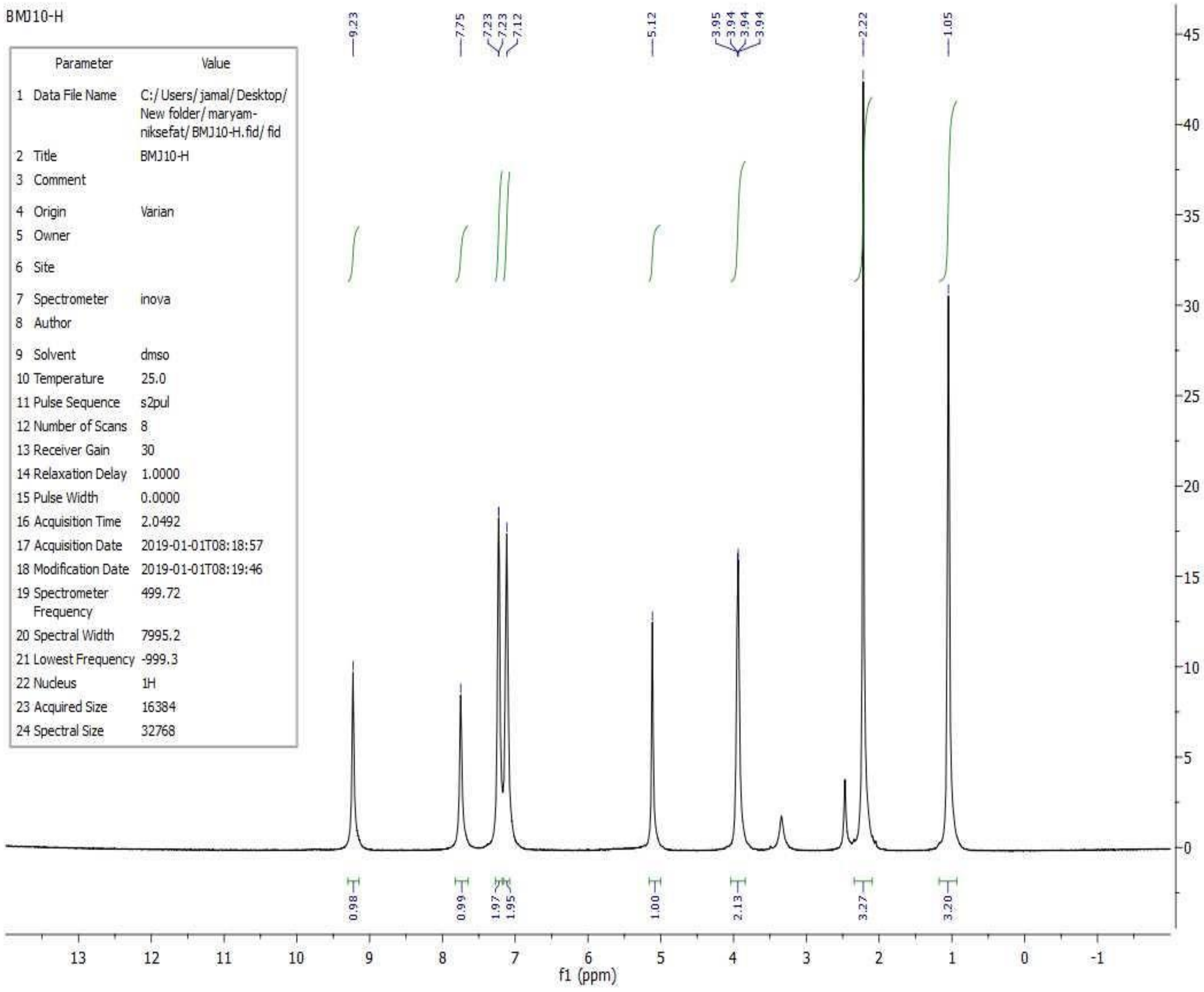

Figure S5. ^{1}H NMR spectrum of the product **4j**.

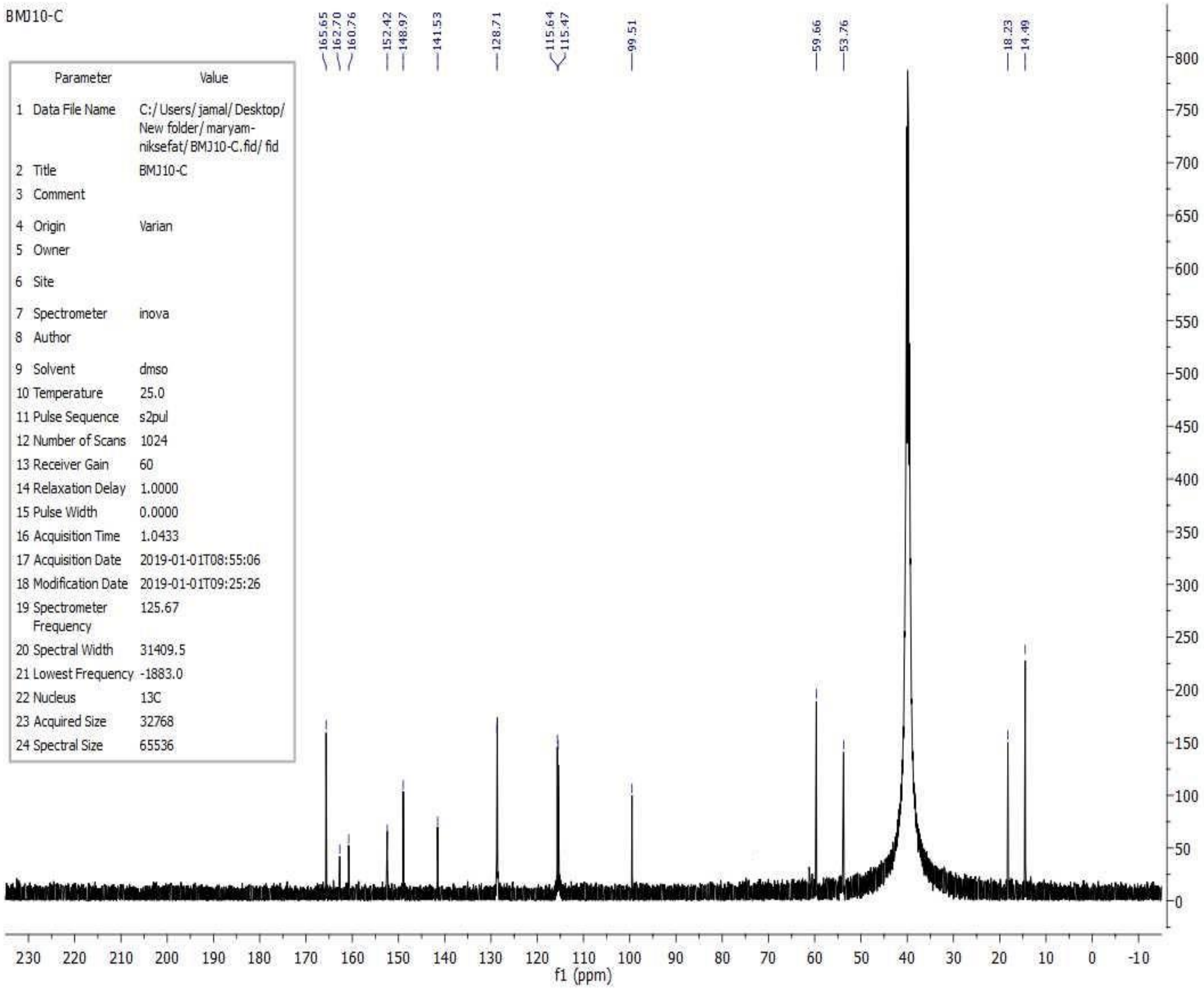

Figure S6. ^{13}C NMR spectrum of the product **4j.**

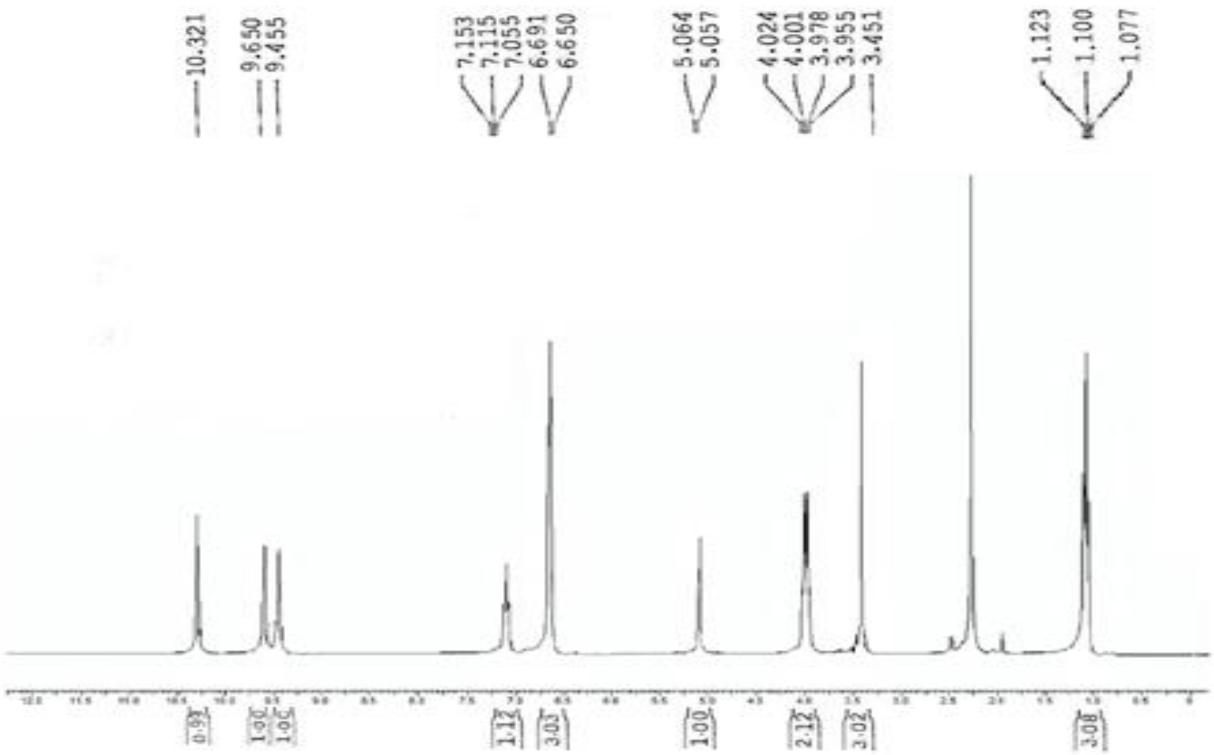

Figure S7. ¹H NMR spectrum of the product **4r**.

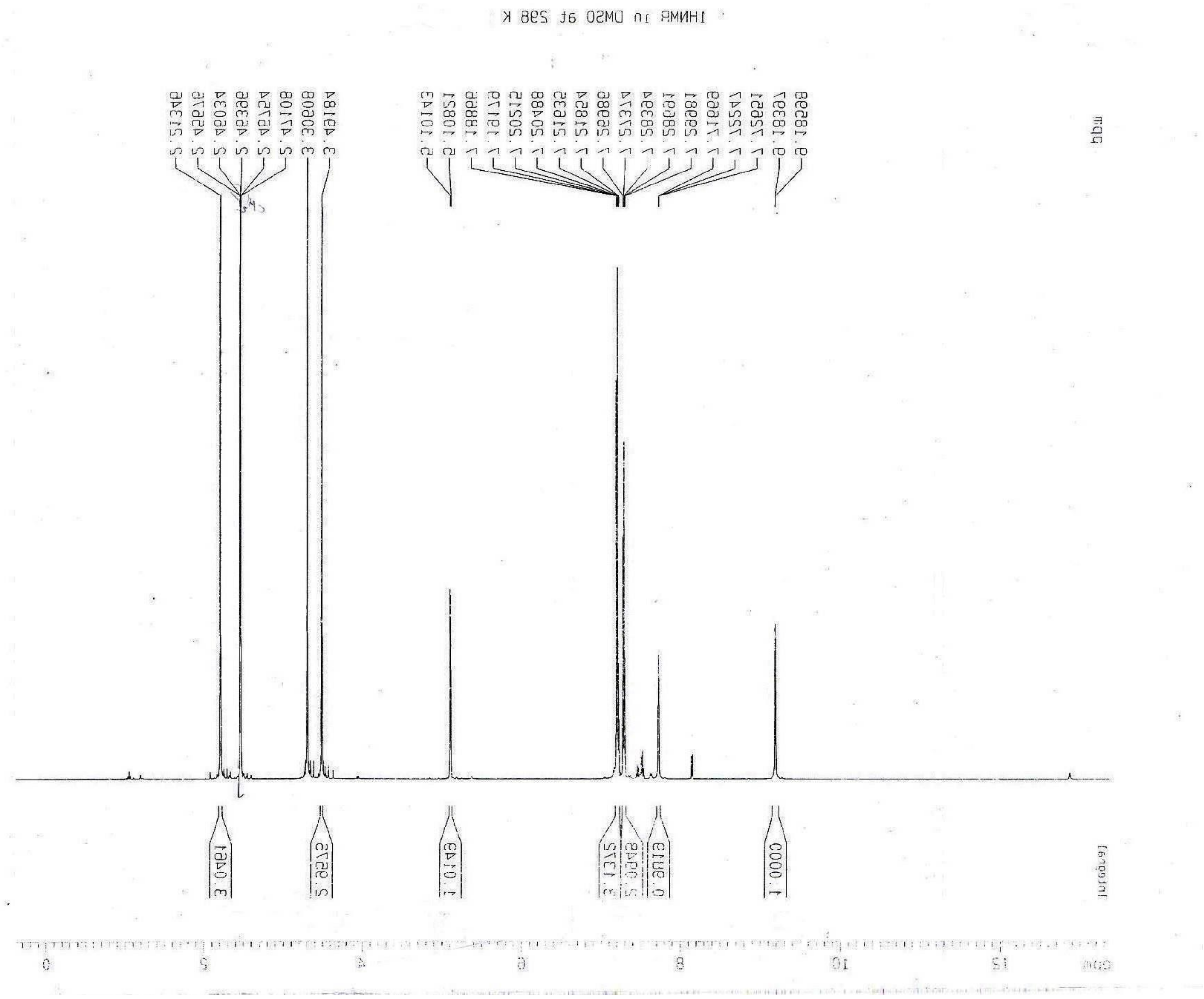

Figure S8. ^{1}H NMR spectrum of the product **4s**.

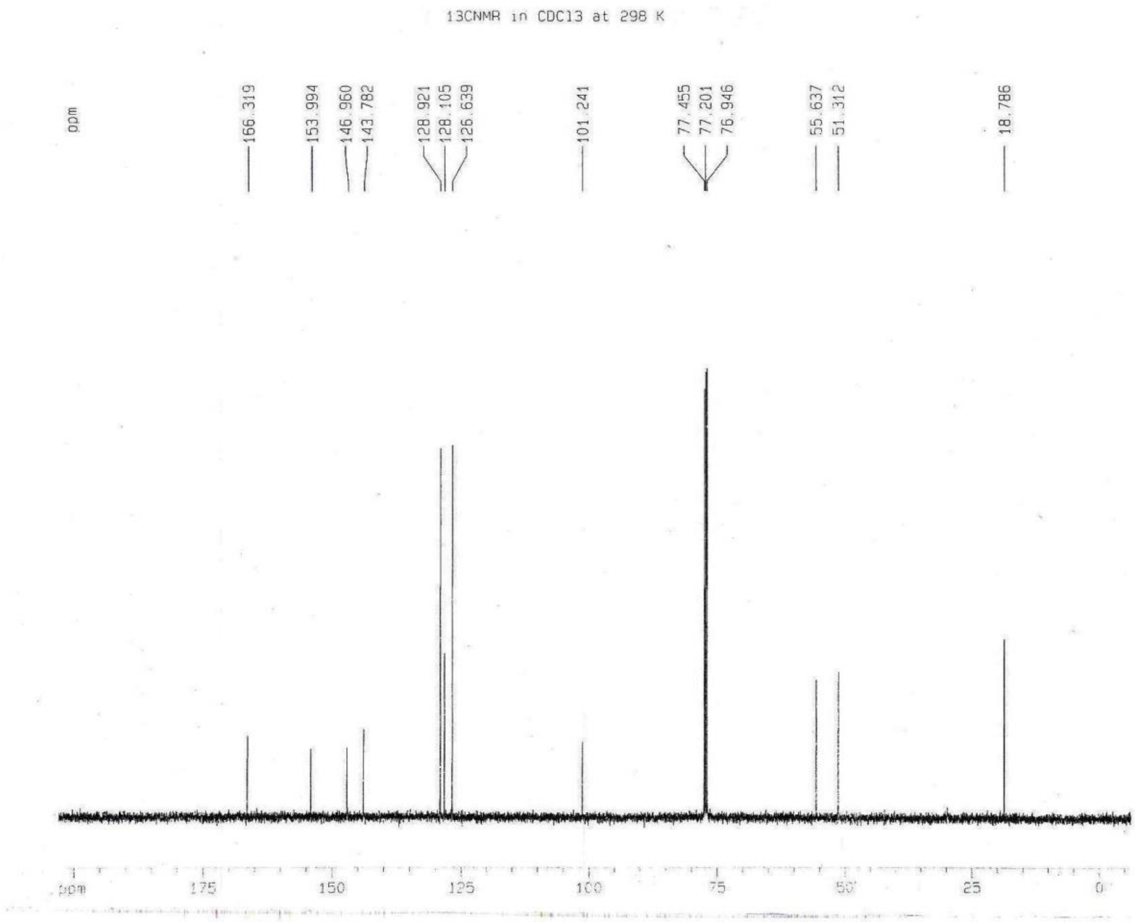

Figure S9. ^{13}C NMR spectrum of the product **4s**.

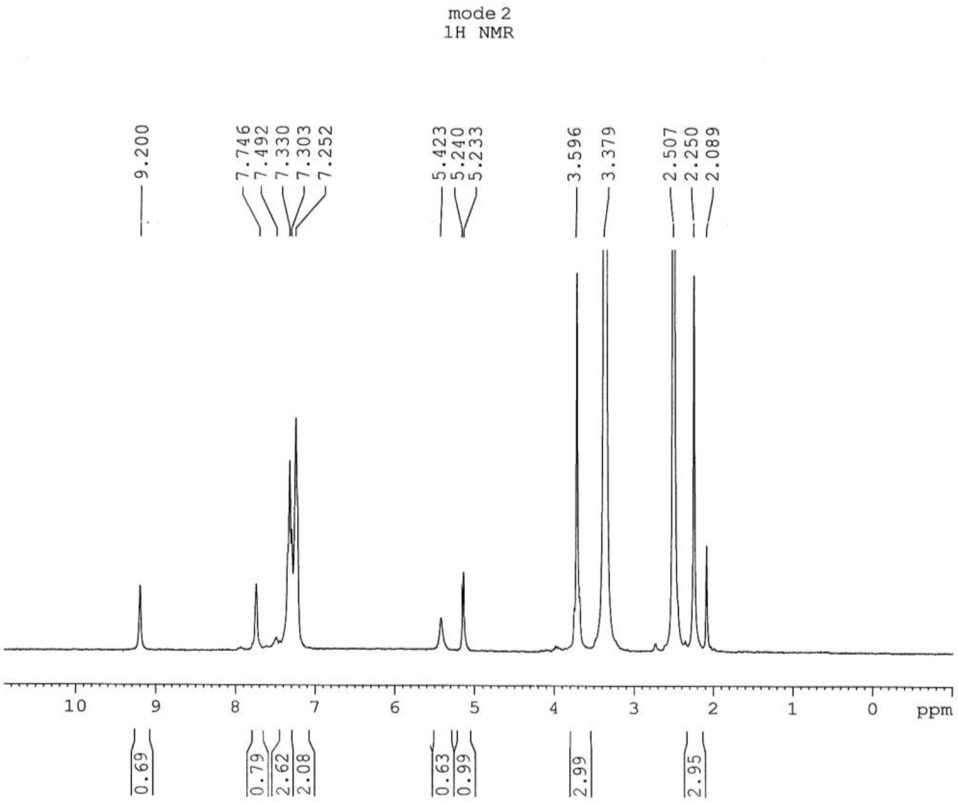

Figure S10. ^{1}H NMR spectrum of the product **4t**.

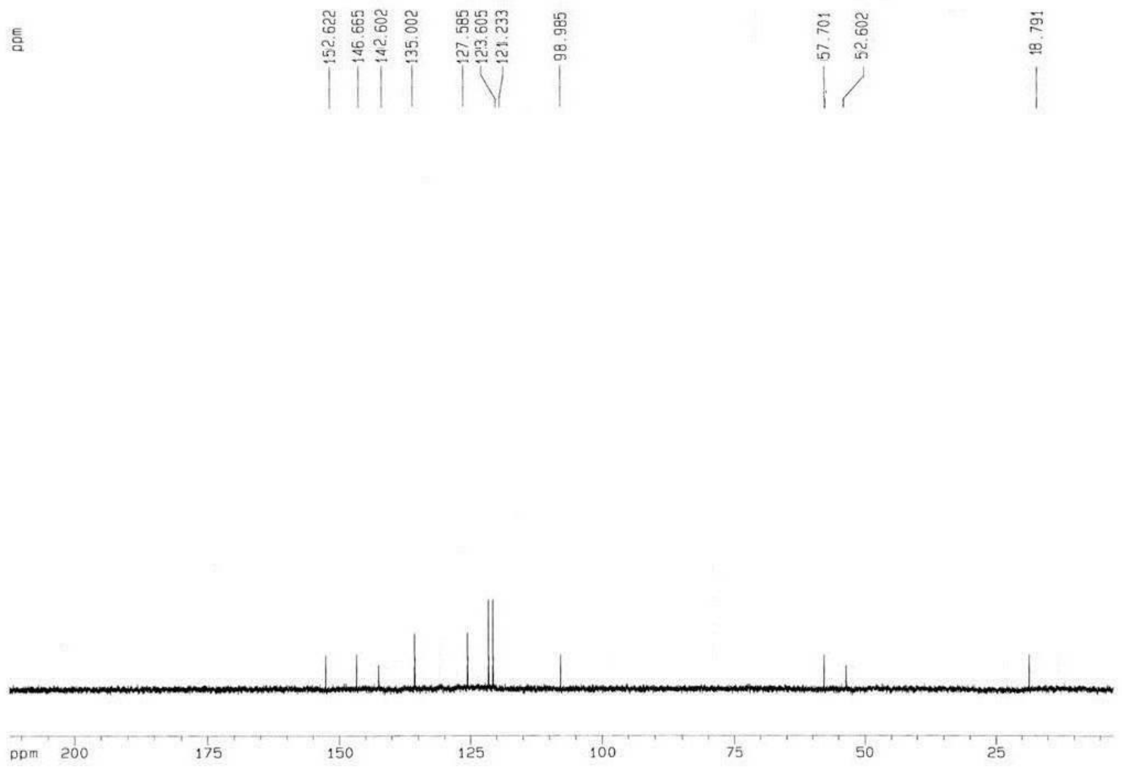

Figure S11. ^{13}C NMR spectrum of the product **4t**.

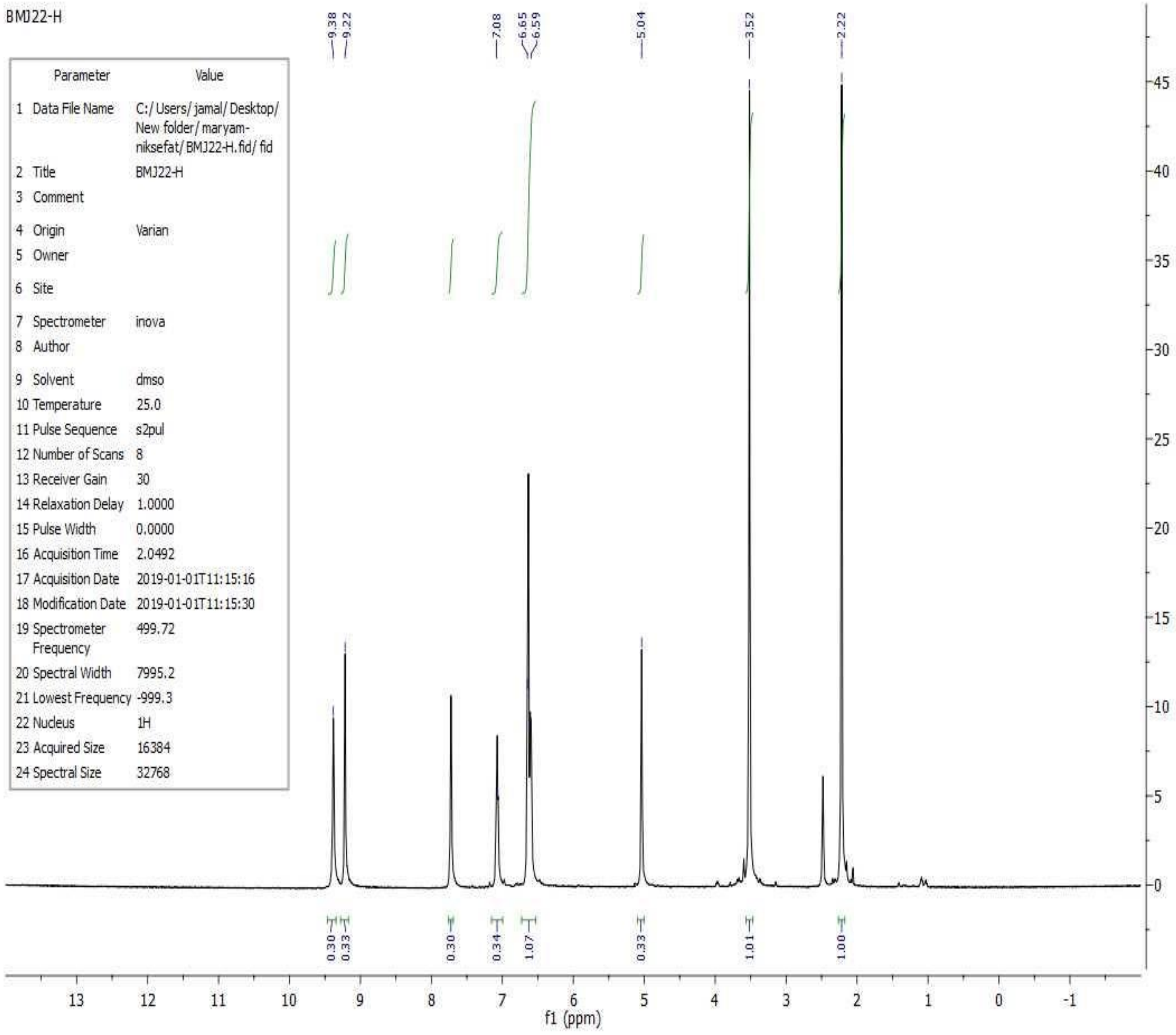

Figure S12. ^{1}H NMR spectrum of the product **4v**.

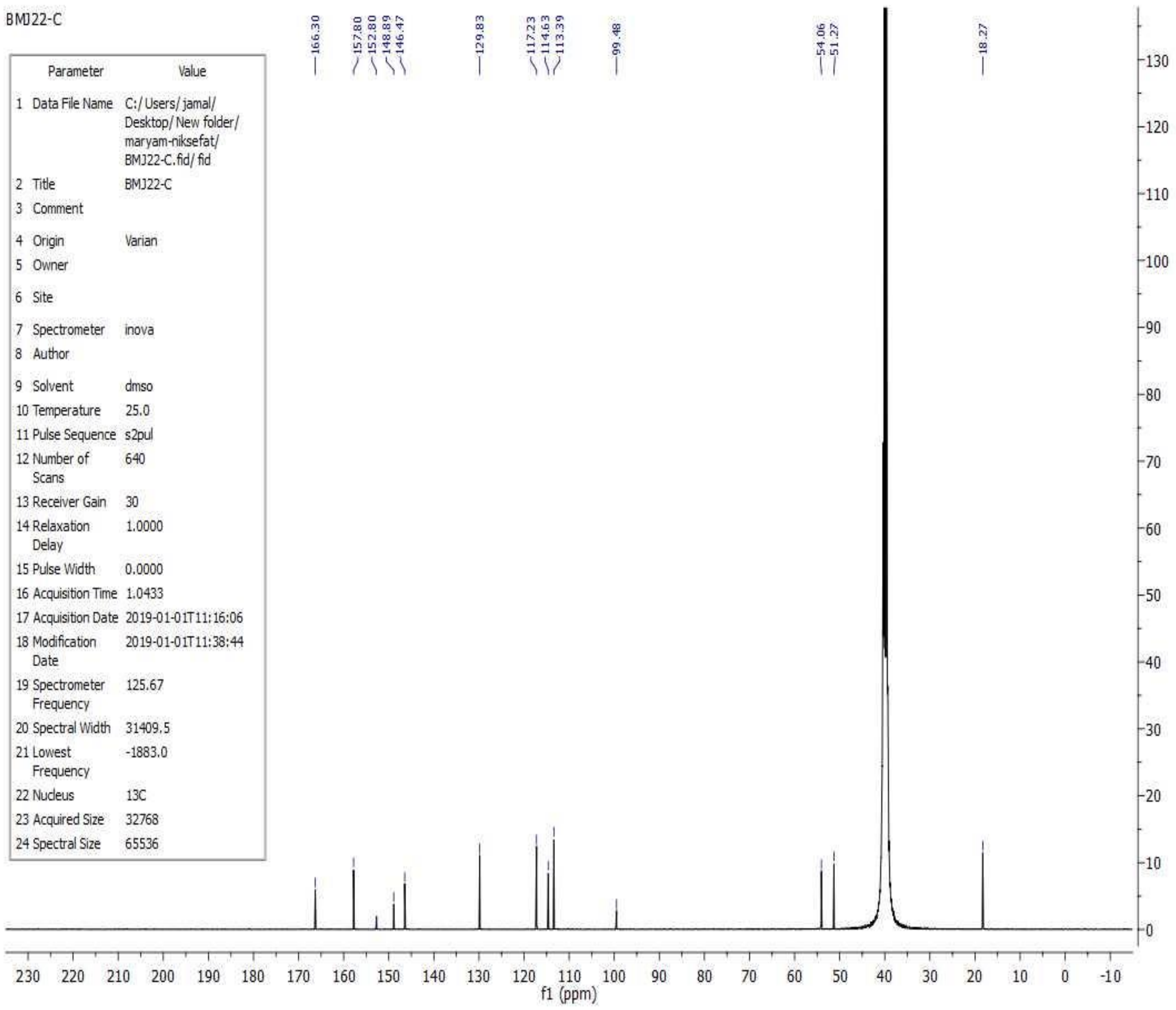

Figure S13. ^{13}C NMR spectrum of the product **4v.**
